# Automated identification of sequence-tailored Cas9 proteins using massive metagenomic data

**DOI:** 10.1038/s41467-022-34213-9

**Published:** 2022-10-29

**Authors:** Matteo Ciciani, Michele Demozzi, Eleonora Pedrazzoli, Elisabetta Visentin, Laura Pezzè, Lorenzo Federico Signorini, Aitor Blanco-Miguez, Moreno Zolfo, Francesco Asnicar, Antonio Casini, Anna Cereseto, Nicola Segata

**Affiliations:** 1 Department of Computational, Cellular and Integrative Biology, University of Trento, Trento, Italy; 2 Alia Therapeutics, Trento, Italy; 3 Present Address: Shmunis School of Biomedicine and Cancer research, Tel Aviv University, Tel Aviv, Israel

**Keywords:** Biotechnology, Computational biology and bioinformatics, CRISPR-Cas9 genome editing

## Abstract

The identification of the protospacer adjacent motif (PAM) sequences of Cas9 nucleases is crucial for their exploitation in genome editing. Here we develop a computational pipeline that was used to interrogate a massively expanded dataset of metagenome and virome assemblies for accurate and comprehensive PAM predictions. This procedure allows the identification and isolation of sequence-tailored Cas9 nucleases by using the target sequence as bait. As proof of concept, starting from the disease-causing mutation P23H in the RHO gene, we find, isolate and experimentally validate a Cas9 which uses the mutated sequence as PAM. Our PAM prediction pipeline will be instrumental to generate a Cas9 nuclease repertoire responding to any PAM requirement.

## Abstract (web summary)

Cas9 proteins require a target-adjacent sequence, the PAM, in order to cleave DNA. Here the authors develop a pipeline to accurately predict PAM sequences in order to facilitate the identification of Cas9s targeting specific sequences, including mutations.

## Introduction

Repositioning CRISPR-Cas systems from prokaryotes to mammalian cells boosted genome editing applications in the clinic^1^. Yet, this technology is still limited by major constraints, mainly related to the reduced number of prokaryotic CRISPR-Cas systems active in mammalian cells which hardly respond to the complexity of gene therapy applications. PAM sequences^2^, necessary for nuclease recognition and activity, are key in this context, as they dictate the compatibility of each CRISPR-Cas tool towards specific genomic target sites. Molecular engineering significantly improved Cas9 properties for genome editing including relaxation of PAM requirements^3^, but this approach impacts the activity of the nucleases and still does not respond to all PAM sequences needed to tackle disease-causing mutations^4^. On the other hand, the rich natural PAM-Cas9 diversity^5^ remains largely unexplored and its efficient exploitation relies on the availability of comprehensive databases and accurate computational Cas9 discovery and PAM prediction tools.

To explore the natural PAM-Cas9 diversity, the pre-determination of the PAM requirements is the crucial step. However, available methods to predict Cas9 PAMs present several limitations (Supplementary Table 1) preventing their effective exploitation: CASPERpam^6^ has very low accuracy (10% functional predictions^7^); SPAMALOT^8^ does not include any spacer match filtering or consensus processing step and its source code is not publicly available; the method described by Vink et al.^9^ associates PAM predictions with clusters of CRISPR repeats, making it not suited to generate predictions for single Cas9 proteins; CRISPRTarget^10^ is only available as a Web service and cannot be automatically interrogated for large sequence repositories; Spacer2PAM^7^ can be applied to predict PAMs of single Cas9 proteins but its low accuracy (45% functional predictions^7^) still impairs comprehensive analyses.

**Fig. 1:**
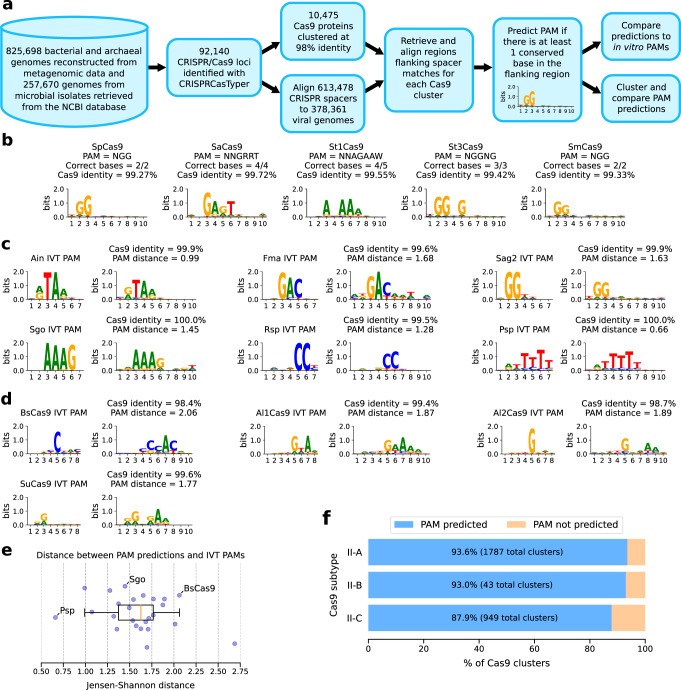
A highly reliable PAM prediction algorithm. **a** Schematic description of the pipeline used to generate PAM predictions. **b** Predicted PAM for Cas9 proteins with experimentally verified PAM. Sequence identity (%) between the reported Cas9 and the Cas9 identified in our dataset is also shown. **c** In vitro PAMs (left logos) and predicted PAM (right logos) for selected Cas9 variants from Gasiunas et al.^19^ (additional orthologs are in Supplementary Fig. 2). A distance measure between predicted and verified PAMs is also shown. **d** In vitro and predicted PAMs (left and right logo respectively) for selected Cas9 variants from our dataset. **e** Boxplot of distance between in vitro and predicted PAMs for all tested orthologs. Most orthologs have a low (<2 bits) PAM distance. Central line, median; box limits, upper and lower quartiles; whiskers, ×1.5 interquartile range; *n* = 25 independent PAMs. **f** Fraction of Cas9 clusters with more than 10 mapped spacers and a predicted PAM, for each Cas9 subtype.

Here we demonstrate that the interrogation of massive metagenomic datasets combined with an improved computational method allows the identification of a vast number of unreported Type II systems and their respective PAM requirements (Fig. 1a).

## Results

### A PAM prediction pipeline applied to massive metagenomic data

From a compendium of 825,698 bacterial and archaeal genomes reconstructed via metagenomic assembly of human, host-associated, and non-host associated environmental microbiomes (see Methods section: Catalog of reference and metagenomic-assembled genomes) and 257,670 genomes from microbial isolates retrieved from the NCBI database, we identified the sequence of 92,140 CRISPR-Cas9 loci. Our pipeline then performed Cas9 proteins clustering at multiple sequence identity levels (from 95% to 100%) and applied a PAM-prediction procedure on each cluster. For predicting the PAMs, we first identified sequences adjacent to protospacers by aligning 613,478 unique spacers to phage genomes of the human microbiome: 142,809 viral genomes from the Gut Phage Database^11^, 189,680 from the Metagenomic Gut Viral catalog^12^ and 45,872 from de-novo assembled gut phages from highly enriched viromes as profiled via ViromeQC^13^ (see Methods section: Viral genomes retrieval from highly enriched viromes). In this procedure we mostly focused on human-associated bacterial and viral genomes as they are largely overrepresented in our dataset, and in particular the human gut has been sufficiently densely sampled to permit multiple reliable host-virus associations via spacer matching (Supplementary Fig. 1). Only full-length, near-perfect matches were retained (at most 4 nucleotide variations), resulting in a total of 39,109,402 putative protospacers. Cas9 clusters with less than 10 mapped spacers were discarded to retain only highly reliable PAM predictions. Upstream and downstream sequences flanking the matches, up to 30 nt, were retrieved. For each Cas9 cluster, sequences flanking the same spacer were realigned and the multiple sequence alignment was collapsed into a single consensus flanking sequence. Nucleotide frequencies in the consensus flanking sequences were computed and represented as sequence logos. A PAM was predicted for a Cas9 cluster if there was at least one conserved position in either the upstream or the downstream flanking sequence (see Methods section: PAM prediction). Repeating the PAM-prediction procedure on the different Cas9 clustering identity thresholds, we concluded that the highest reliability was obtained at 98% identity clustering (see Methods section: PAM comparisons and hierarchical clustering). At this clustering stringency, we obtained PAM predictions for 2546 out of 2779 Cas9 clusters (representing 61,095 Cas9 sequences) with more than 10 mapped spacers (91.6%).

### Validation of predictions via in vitro determined PAMs

To validate our approach and predictions, we searched our dataset for gene sequences coding for proteins with high sequence identity (>98%) to previously characterized Cas9s: SpCas9^14^, SaCas9^15^, St1Cas9^16^, St3Cas9^17^, and SmCas9^18^. For these Cas9s we obtained sequence predictions corresponding to the described PAMs (Fig. 1b). We further tested our method by cross checking the PAM predictions obtained with our pipeline with the sequences experimentally identified and recently reported and characterized by Gasiunas et al.^19^ Of the 79 Cas9s reported, 21 could be used for the evaluation here as they had a close ortholog in our dataset (>98% identity), and for them we confirmed the accuracy of our prediction strategy by obtaining PAM logos with high identity (assessed by Jensen-Shannon distance on nucleotide frequencies, see Methods section: PAM comparisons and hierarchical clustering) with the sequences determined experimentally (Fig. 1c and Supplementary Fig. 2). Overall, 18 out of 21 (85%) PAM predictions generated by our method were correct and the remaining 15% were partial predictions with at least one base correctly identified. Our method exhibited a much higher prediction accuracy compared to Spacer2PAM^7^ (Supplementary Fig. 3), which outperforms both CRISPRTarget and CASPERpam^7^ and was therefore chosen as the gold standard for the comparison.

To further test experimentally the reliability and potential of the PAM prediction pipeline in expanding the Cas9 toolbox, we searched for Cas9 candidates using parameters favoring the identification of functionally active enzymes (with preserved domain structures and located in complete CRISPR-Cas loci) and with reduced molecular size (<1100 amino acids), thus potentially more convenient for genome editing applications. We identified four Cas9s never described before from poorly characterized species (Supplementary Fig. 4) and predicted their PAM logos which were subsequently experimentally validated through an in vitro assay^20^. Results demonstrated a very close identity between in silico and in vitro results as indicated by the small distance (<2 bits for 3 out of 4 Cas9 variants) between predicted and experimentally determined PAMs (Fig. 1d-e), thus further confirming the accuracy and the potential of this PAM prediction pipeline. Overall, our method allowed PAM prediction for the vast majority of Cas9 proteins identified in our repository with 10 or more mapped spacers, across all Cas9 subtypes (93.6% for A, 93.0% for B and 87.9% for C; Fig. 1f).

### Cas9 PAMs contain non-random nucleotide combinations

**Fig. 2:**
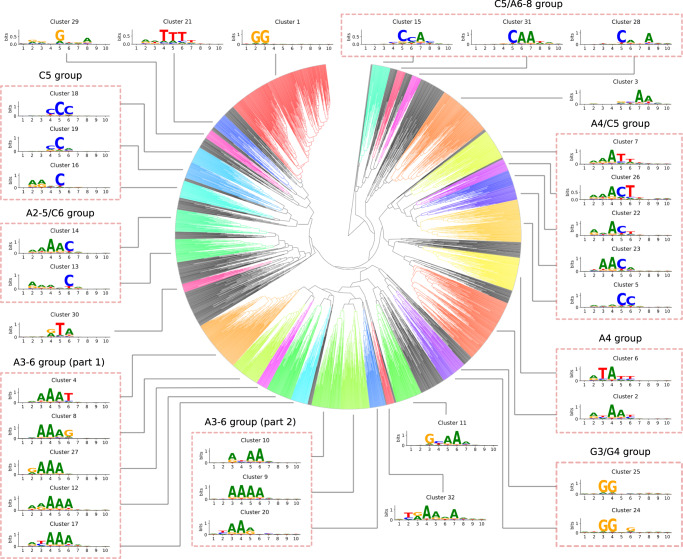
PAM hierarchical clustering. Hierarchical clustering tree generated from pairwise PAM distances, with annotated PAM groups and consensus PAM for each cluster. Different groups of PAMs with conserved bases in specific positions can be identified.

We then applied our PAM predictor to the metagenomically extended set of 2,546 Cas9 protein families (98% identity clustering) to identify all PAM requirements and explore whether specific PAM groups may exist. Hierarchical clustering on pairwise distance of the predicted PAMs retrieved 32 groups with at least 20 members (see Methods section: PAM comparisons and hierarchical clustering). For each PAM group, a consensus PAM was generated (Fig. 2). Interestingly, the most prevalent PAM sequences represent only a small fraction of all possible PAMs. Therefore, even though the PAM variability is high for type II Cas9^9^, only definite combinations of nucleotides were identified.

We further evaluated whether there might be an association between the PAM groups identified in Fig. 2 and specific Cas9 subtypes. After generating a phylogenetic tree of the identified Cas9, we found that almost every PAM group was associated with specific clades of Cas9 proteins (Supplementary Fig. 5), thus suggesting a non-random organization of PAM recognition sequences. For instance, the most abundant PAM group (NGG) was found in a specific branch of type II-A and in almost all type II-B Cas9s.

### Predicted PAMs have the potential to target most pathogenic mutations

**Fig. 3:**
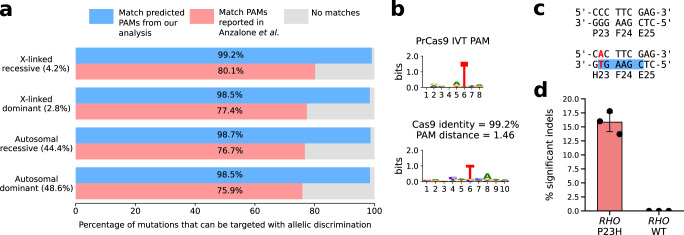
Fraction of ClinVar pathogenic mutations matching predicted PAMs and isolation of an unreported Cas9 ortholog tailored for a disease-causing mutation. **a** Percentages of mutations in ClinVar that could potentially be targeted with allelic discrimination by Cas9 proteins identified by our analysis and by Cas nucleases already used in the genome editing field^23^, divided by mode of inheritance. **b** The predicted PAM of PrCas9 matches the in vitro PAM logo. Preference for bases other than T in position 6 can be observed using a PAM heatmap (Supplementary Fig. 6), resulting in the NRVNRT PAM where V = A, C or G; R = G or A. **c** Wild-type (WT) and P23H *RHO* alleles, showing the mutation resulting in the P23H substitution (in red) and matching PAM on the mutated allele (highlighted in blue). **d** Editing activity (% significant indels obtained by TIDE analysis, see Methods: Cell culture and indels analysis) in *RHO* wild-type or carrying the P23H mutation (*n* = 3 each, mean ± SD, biologically independent samples).

To prove the potential of this prediction pipeline in genome editing applications we turned to a specific class of mutations that are associated with autosomal dominant genetic diseases due to an induced gain of function to the mutated product. These mutations can be neutralized through CRISPR-Cas knockouts but the lack of allelic discrimination due to various grades of sgRNA mismatch tolerance by Cas9^21^ limits the effective targeting of the mutated allele. Conversely, since PAM sequences are stringent requirements for Cas9 activity, using PAMs matching the mutated bases would allow a specific target separation between the mutated and the wild-type alleles. Consequently, a paramount application of our PAM prediction pipeline is the identification of uncharacterized Cas9s recognizing PAM sequences generated by pathogenic mutations to offer specific targeting options for the mutated allele with a highly secured allelic discrimination. By interrogating the ClinVar database^22^ for mutations corresponding to PAMs associated with Cas9s from our metagenomic analysis, we found that a large fraction of pathogenic mutations (98.6% of 89,751 substitutions and small indels with known mode of inheritance) are included in at least one of the identified PAMs, thus having the potential to provide allelic discrimination, with 48.6% of them being autosomal dominant alterations (Fig. 3a). Conversely, we estimated that only 76.1% of pathogenic mutations can be potentially targeted with allelic discrimination by Cas nucleases already used in the genome editing field^23^, thus the PAM diversity identified by our analysis provides a nearly complete coverage of pathogenic mutations that was previously lacking.

### Identification of a tailored Cas9 targeting the *RHO* P23H mutation

As a proof of concept for the potential of our PAM prediction method, we chose a specific dominant-negative mutation, the P23H mutation (c.68 C > A) in the *rhodopsin* (*RHO*) gene^24^, which is the most common mutation causing *RHO*-dependent retinitis pigmentosa^25^. Looking for a Cas9 that could uniquely target the P23H mutation, we identified PrCas9 (Supplementary Table 2), a Cas9 found in an unclassified species from the Proteobacteria phylum, which has a predicted PAM N_5_T (Fig. 3b), where the T in the PAM is generated by the P23H mutation in *RHO* (CGAAG**T**, wild-type sequence CGAAGG, Fig. 3c). We experimentally validated in vitro the PrCas9 PAM preference (PAM NRVNRT, Fig. 3c and Supplementary Fig. 6) and tested its editing activity in mammalian cells with an EGFP disruption assay, generating nearly 50% EGFP disrupted cells (Supplementary Fig. 7). We then assessed its efficiency and specificity towards *RHO* P23H mutation by co-transfecting cells with PrCas9 and the same sgRNA targeting RHO together with a plasmid over-expressing either the RHO WT or the RHO P23H minigene. We obtained up to 15.8% indels at the *RHO* P23H locus and the complete absence of indels in the wt sequence, thus demonstrating the efficacy of the selected Cas9 in targeting the *RHO* specific mutation in mammalian cells (Fig. 3d).

## Discussion

By interrogating an extended metagenomic repository with an improved computational approach, we identified a large variety of previously unreported Cas9 nucleases accompanied by their identified PAM requirements. The PAM prediction pipeline here developed showed overall a high level of reliability and better performance than methods so far developed. As more and more metagenomic data will be available the remaining limited differences between predicted and in vitro determined PAMs can be further reduced: mapping spacers to the wrong viral sequence will be less likely with curated and expanded viral databases; higher numbers of mapped spacers will allow increasing the current minimum number of ten mapped spacers and generate more reliable consensus predictions; finally, more sequences will also minimize the risk to be impacted by mutations in protospacer adjacent sequences introduced in order to evade CRISPR-Cas mediated immunity^26^. The difference between the motif recognized by the spacer acquisition machinery (SAM) and the one recognized by the target interference effector (TIM) that has been observed for some CRISPR-Cas systems^27^ is likely not contributing to the discrepancies between predicted and in vitro determined PAMs, since Cas9 is directly involved in spacer acquisition^28^.

Our analysis revealed that PAM sequences follow defined nucleotide patterns which are associated with specific Cas9 subtypes and have the potential to overlap with 98.6% of the pathogenic mutations reported in ClinVar^22^. The precise PAM prediction driven by a specific sequence-mutation query allows the identification of tailored Cas9s, such as PrCas9 targeting the P23H *RHO* mutation. We expect that as reported before^15^ several Cas9 variants from the sequence repository here reported will not be active in mammalian cells. Nonetheless, the large variety of orthologs identified in our repository combined with optimized computational pipeline will highly enhance the efficiency of Cas mining protocols. This approach opens to the expansion of the genome editing toolbox with mutation-tailored nucleases and supports the strategy of an application-specific search for suitable natural prokaryotic genome editing tools requiring minimal or no engineering.

## Methods

### Catalog of reference and metagenomic-assembled genomes

The catalog of bacterial and archaeal genomic sequences used in this work was retrieved from: (i) 257,670 publicly available isolated sequences from the NCBI database^29^ (available as of January 2021), (ii) 771,529 metagenome-assembled genomes (MAGs) from the Blanco-Miguez et al. study^30^, and (iii) 54,169 additional MAGs obtained with a validated assembly-based pipeline similarly to Pasolli et al.^31^ For retrieving these 54,169 additional MAGs, 8487 metagenomic samples (Supplementary Table 3) were assembled using metaSPAdes^32^ if paired-end metagenomes were available, and MEGAHIT^33^ otherwise. In both cases, default parameters were used. Contigs longer than 1500 nucleotides were binned into MAGs using MetaBAT2^34^.

### Viral genomes retrieval from highly enriched viromes

A total of 45,872 viral genomes were metagenomically assembled from 3044 Human Gut virome datasets as described previously^35^. In brief: the efficacy of viral enrichment in each virome was evaluated with ViromeQC^13^ (version 1.0). A total of 255 samples had an enrichment higher than 50X and were retained as highly viral samples. Reads were preprocessed with TrimGalore (version 0.4.4)^36^ to remove low quality and short reads (parameters:–stringency 5–length 75–quality 20–max_n 2–trim-n). Reads aligning to the human genome hg19 were also removed with Bowtie2 (version 2.4.1)^37^. High quality reads were assembled into contigs with metaSPAdes (version 3.10.1)^32^ (k-mer sizes: -k 21,33,55,77,99,127), or MEGAHIT (version 1.1.1)^33^.

To reduce non-viral contaminants, we removed contigs that mapped to microbial genomes by using the collection of MAGs from Pasolli et al.^31^. Only contigs that were (a) longer than 1500 bp; (b) found within the same microbial species-level genome bin in <30 metagenomes; and (c) found in the unbinned assembled fraction of more than 20 metagenomes, were retained. Contigs from (i) the remaining non-highly enriched viromes, and (ii) from the human gut metagenomes used in Pasolli et al.^31^, and that were similar to a potentially highly enriched viral genome, were also mapped against the unbinned contigs of Pasolli et al. with mash (version 2.0)^38^. Contigs with a distance lower than 10% (*p*-value ≤ 0.05) were retained. Finally, we selected 699 complete viral genomes from RefSeq, release 99^39^ by selecting genomes that could be found in at least 20 samples within the unbinned contigs of Pasolli et al.^31^. All mappings were performed with blastn (version 2.6.0)^40^ identity >80%, aln. len. >1000 bp). Contigs were clustered at 95% identity with VSEARCH^41^ with each cluster needing to contain at least one contig originating from highly enriched viromes.

### PAM prediction

CRISPRCasTyper (version 1.5.0, default parameters)^42^ was used to identify 131,941 CRISPR-Cas loci. This tool identifies CRISPR-Cas loci in MAGs searching for CRISPR arrays and cas operons in close proximity to each other (up to 10,000 bp). Loci containing Cas9 proteins shorter than 950 aa were excluded from the analysis. The resulting 92,140 Cas9 proteins were clustered at 100, 99, 98, 97, 96, and 95% identity using usearch (version 11.0.667)^43^ resulting in 27,062, 14,332, 10,475, 8568, 7538, and 6898 clusters respectively.

For each Cas9 cluster, spacers in CRISPR arrays adjacent to *cas* genes were retrieved and oriented according to the orientation of *cas1*, *cas2*, and *cas9* genes. In total, 613,478 spacers were retrieved from CRISPR arrays and were aligned to 366,233 viral genomes (142,809 from Gut Phage Database^11^, 189,680 from Metagenomic Gut Virus catalog^12^ and 45,872 from *de-novo* assembled gut phages from highly enriched viromes) using blastn (version 2.5.0)^40^ to identify putative protospacers. Matches with more than 4 mismatches or gaps were filtered out. For each Cas9 clustering level, clusters with less than 10 mapped spacers were discarded, resulting in 7177 (26.52%), 3908 (27.27%), 2779 (26.53%), 2169 (25.32%), 1814 (24.06%), and 1594 (23.11%) clusters. Since the orientation of CRISPR arrays is unknown, both upstream and downstream flanking sequences, up to 30 nt, were retrieved for each putative protospacer. For each Cas9 cluster, protospacer and their flanking sequences, found using the same spacer, were aligned to each other using MUSCLE (version 3.8.31)^44^ and the alignment was collapsed into a single consensus sequence by taking the most frequent base at each position and discarding columns composed mostly (>50%) of gaps. Spacers were aligned exactly to the consensus sequence to define up- and downstream regions, which were then used to compute nucleotide frequencies and generate sequence logos using Logomaker (version 0.8)^45^.

For each Cas9 cluster, a PAM was considered predicted if there was at least one highly conserved base in only one of the two flanking regions (the PAM can be either upstream or downstream, not both). We defined a highly conserved base as a position in the logo with more information than the maximum between 1 bit and the third quartile plus 1.5 times the interquartile range of the distribution of information in both flanking sequences (i.e. the conserved position is an outlier with at least 1 bit of information). For each clustering level, a PAM was predicted for 6758 (94.16%), 3622 (92.68%), 2546 (91.62%), 1944 (89.63%), 1601 (88.26%), and 1387 (87.01%) clusters with more than 10 mapped spacers.

### tracrRNA identification

tracrRNA sequences of the previously uncharacterized Cas9 orthologs were identified computationally, searching for sequences starting with a putative anti-repeat and ending with a Rho-independent transcription terminator (RIT). Putative anti-repeats were identified aligning CRISPR repeats to sequences flanking the CRISPR-Cas locus (up to 1000 nt) using blastn (version 2.5.0)^40^ and RITs were predicted using RNIE^46^.

### In vitro PAM determination

In vitro PAM evaluation of the previously uncharacterized Cas9 orthologs was performed according to the protocol from Karvelis et al.^20^. In brief: for each Cas9 ortholog the human codon optimized version of its coding sequences was ordered as a synthetic construct (Genscript) and cloned into an expression vector for in vitro transcription and translation (IVT) (pT7-N-His-GST- Thermo Fisher Scientific). Reaction was performed according to the manufacturer protocol (1-Step Human High-Yield Mini IVT Kit - Thermo Fisher Scientific). The Cas9-guideRNA RNP complex was assembled by combining 20 μL of the supernatant containing soluble Cas9 protein with 1 μL of RiboLock RNase Inhibitor (Thermo Fisher Scientific) and 2 μg of guide RNA. The Cas9-guideRNA complex obtained was diluted 1:10 as described in Karvelis et al.^20^ and used to digest 1 μg of a plasmid (p11-lacY-wtx backbone - Addgene #69056) containing an 8-nucleotide randomized PAM sequence flanking the gRNA target. Digestion reaction was incubated for 1 h at 37 °C. A double-stranded DNA adapter^20^ was then ligated to the DNA ends generated by the targeted Cas9 cleavage and the final ligation product was purified using a GeneJet PCR Purification Kit (Thermo Fisher Scientific). One round of a two-step PCR (Phusion HF DNA polymerase - Thermo Fisher Scientific) was performed as described in Karvelis et al.^20^ to enrich the sequences that were cut using a set of forward primers annealing on the adapter and a reverse primer designed on the plasmid backbone downstream of the PAM (Supplementary Table 4). A second round of PCR was performed to attach the Illumina indexes and adapters (Nextera XT Index Kit v2 Set A). PCR products were purified using Agencourt AMPure beads in a 1:0.8 ratio.

The generated library was analyzed with a 71-bp single read sequencing, using a flow cell v2 micro, on an Illumina MiSeq sequencer. PAM sequences were extracted from Illumina MiSeq reads and used to generate PAM sequence logos. PAM heatmaps^47^ were used to display PAM enrichment, computed dividing the frequency of PAM sequences in the cleaved library by the frequency of the same sequences in a control uncleaved library.

### PAM comparisons and hierarchical clustering

Differences between PAM sequences were quantified using the Jensen-Shannon distance (defined as the square root of the Jensen-Shannon divergence)^48^. This distance quantifies the difference between predicted and experimentally validated PAMs by comparing the bit-score of each base at every position in both PAM. As shown in Fig. 1e, predictions that closely match experimentally validated PAMs have a distance lower than 2 bits.

To find the optimal Cas9 clustering identity level, the Jensen-Shannon distance between predicted and in vitro determined PAMs was computed for 16 Cas9 orthologs characterized by Gasiunas et al.^19^ having a prediction at each clustering level. PAM predictions resulting from the 98% identity Cas9 clustering showed the lowest median distance and were therefore chosen for subsequent analyses (Supplementary Table 5).

Spacer2PAM^7^ predictions were generated using unique spacer sequences derived from the 98% identity Cas9 clustering. Spacer2PAM (version 0.0.0.9000) was run with default parameters, except the e-value threshold which was set to 0.01. A two-sided Welch’s *t*-test was used to assess the statistical significance (*p*-value = 1.5e-5) of the difference between the Jensen-Shannon distance of in vitro determined PAMs and predictions generated by Spacer2PAM and by our method.

An all-to-all PAM prediction distance matrix was computed and hierarchical clustering was performed to generate PAM groups, using usearch (version 11.0.667, parameters -cluster_aggd -id 0.6 -linkage avg)^43^. Consensus PAMs for each cluster were generated using sequences adjacent to protospacers of all cluster members.

### PAM groups association with Cas9 phylogenetic tree

Cas9 proteins with a predicted PAM (98% identity clustering) were aligned using mafft (version 7.490, with parameters–maxiterate 10)^49^ and a phylogenetic tree was built using FastTree (version 2.1.11, with parameters -spr 4 -mlacc 2 -slownni)^50^. Cas9 clades were defined using TreeCluster (version 1.0.3, with parameters -m max_clade)^51^ and a range of distance thresholds (0.3–4). Associations between PAM groups and Cas9 clades were assessed using a two-sided Fisher’s exact test using R 4.1.0, computing p-values by Monte Carlo simulation with 100,000 replicates. *P*-values were adjusted for multiple hypothesis testing using the Benjamini-Hochberg correction and resulted <0.001 for all PAM groups and almost all distance thresholds.

### Identification of PAM-matching mutations in ClinVar

Mutations in the ClinVar database (accessed 6 March 2022)^22^ were filtered to select single nucleotide variants and short indels (10 or less nucleotides) annotated as pathogenic or likely pathogenic and associated with pathologies with known mode of inheritance, for a total of 89,751 mutations. Predicted PAMs resulting from the 98% identity Cas9 clustering were converted to consensus sequences by taking at each position bases with more information than half the threshold used previously to define highly conserved bases, to avoid underestimating the number of non-N bases in the consensus sequence. To compute the fraction of mutations that can be targeted by at least a Cas9 in our databank with allelic discrimination, consensus sequences derived by our analysis and PAMs of Cas nucleases already used in the genome editing field were then aligned exactly to wild-type and mutated alleles.

### Cell culture and indels analysis

HEK293T/17 obtained from ATCC (CRL-11268) were cultured in DMEM supplemented with 10% fetal bovine serum, 2 mM l-Glutamine, 100 U/ml Penicillin, and 100 μg/ml streptomycin (Life Technologies) and incubated at 37 °C and 5% CO_2_ in a humidified atmosphere. Cells tested mycoplasma negative (PlasmoTest, Invivogen). For indels analyses, HEK293T/17 cells were seeded in 24-well plate and transfected after 24 h with 1000 ng pX-PrCas9-sgRNA-RHO-P23H, 50 ng pCMV-TO-RHO-P23H or pCMV-TO-RHO-WT and 50 ng pEGFP-IRES-Puro using TransIT-LT1 transfection reagent (Mirus Bio) according to manufacturer’s instructions. 48 hours post-transfection cells were pool-selected with 1 μg/ml Puromycin and collected after 72 hours. Genomic DNA was obtained from cell pellets using the QuickExtract DNA extraction solution (Lucigen) according to the manufacturer’s instructions. The *RHO* P23 locus was amplified using the HOT FIREPol Multiplex Mix (Solis Biodyne) with primers RHO-TO-F (CAGTGATAGAGATCTCCCTATC) and RHO-int1-R (GAGATAGATGCGGGCTTCCA). PCR amplicons were purified using CleanNGS beads (CleanNA) and Sanger sequenced (Microsynth) using RHO-TO-F primer. Indel levels were evaluated using TIDE^52^.

### Plasmids

A pX330-derived plasmid was used to express the Cas9 orthologs in mammalian cells. Briefly, pX330 (Addgene) was modified by substituting SpCas9 and its sgRNA scaffold with the human codon-optimized coding sequence of the variant of interest and its sgRNA scaffold. The Cas9 variants coding sequences, modified, as described before, by the addition of an SV5 tag at the N-terminus and two nuclear localization signals bpNLS^53^ (1 at the N-term and 1 at the C-term) and human codon-optimized, as well as the sgRNA scaffolds, were obtained as synthetic fragments from either Genscript or Genewiz. Spacer sequences were cloned into the pX-Cas9 plasmids as annealed DNA oligonucleotides containing a variable 20 or 24-nt spacer sequence using a double BsaI site present in the plasmid. The list of spacers sequences used in the EGFP disruption assay and in the evaluation of editing activity against the *RHO* P23H mutation is reported in Supplementary Table 6.

pCMV-TO-RHO-WT plasmid was obtained by cloning the human rhodopsin (*RHO*) gene into the pCDNA5/TO plasmid (Addgene). The *hRHO* gene was PCR-amplified using the primers RHO_gene_F (attaggatccAGAGTCATCCAGCTGGAGCCC) and RHO_gene_R (taatctcgagTGGGGTTTTTCCCATTCCCAGG) from genomic DNA extracted from HEK293T/17 cells using the Phusion high fidelity DNA Polymerase (ThermoFisher Scientific). The P23H mutation was further inserted by site-directed mutagenesis using primers mut-P23H-F (GTGTGGTACGCAGCCaCTTCGAGTACCCACAG) and mut-P23H-R (CTGTGGGTACTCGAAGtGGCTGCGTACCACAC) to generate pCMV-TO-RHO-P23H plasmid. All the oligonucleotides were purchased from Eurofins Genomics.

### Reporting summary

Further information on research design is available in the Nature Research Reporting Summary linked to this article.

## Supplementary information

Supplementary Information

Reporting Summary

## Data Availability

The data that support the findings of this study are available from the material associated with 10.1101/2022.08.22.504593. Genome assemblies from which CRISPR-Cas9 loci were identified are available at the European Nucleotide Archive under accessions PRJNA391943, PRJEB31266, PRJEB31003, PRJEB26432, PRJEB33885. Additional genome assembiles are available at http://segatalab.cibio.unitn.it/data/Pasolli_et_al.html, https://genome.jgi.doe.gov/portal/GEMs/GEMs.home.html, http://segatalab.cibio.unitn.it/data/Manara_et_al.html and https://zenodo.org/record/3631711.

## Code availability

The code used to generate PAM predictions is freely available at: https://github.com/Matteo-Ciciani/PAMpredict. Additional code used to analyze the PAM predictions is freely available at: https://github.com/Matteo-Ciciani/PAM_prediction_analysis.
