# Supplementary material for: Automated identification of sequence-tailored Cas9 proteins using massive metagenomic data
Source: Nat Commun. 2022 Oct 29;13:6474. doi: 10.1038/s41467-022-34213-9 (PMC9617884; doi:10.1038/s41467-022-34213-9)
Supplement: Supplementary file 1 — Supplementary Information [file 41467_2022_34213_MOESM1_ESM.pdf]

Automated identification of sequence-tailored Cas9 proteins using massive metagenomic data

Matteo Ciciani[1], Michele Demozzi[1], Eleonora Pedrazzoli[1], Elisabetta Visentin[1], Laura Pezzè[2], Lorenzo Federico Signorini[1,3], Aitor Blanco-Miguez[1], Moreno Zolfo[1], Francesco Asnicar[1], Antonio Casini[2], Anna Cereseto[1#*], Nicola Segata[1# *]

Affiliations:

[1] Department of Computational, Cellular and Integrative Biology, University of Trento, Italy

[2] Alia Therapeutics, Trento, Italy

[3] Current address: Shmunis School of Biomedicine and Cancer research, Tel Aviv University, Israel

[#] These authors contributed equally to this work

* Correspondence should be addressed to: anna.cereseto@unitn.it and nicola.segata@unitn.it

Supplementary Information

Table of contents

- **Supplementary Figures 1-7**
- **Supplementary Methods**
- **Supplementary Table 1-5**

Supplementary Figure 1

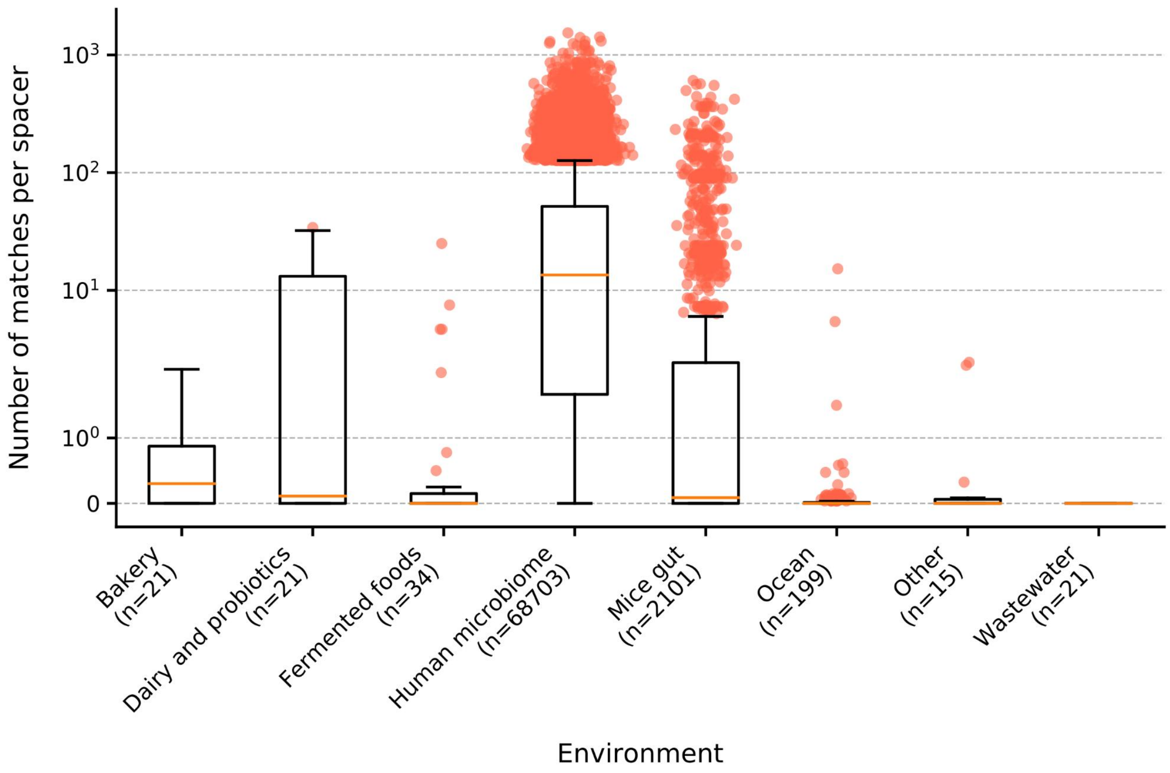

Supplementary Figure 1: Boxplots of the number of matches per spacer for CRISPR-Cas loci identified in metagenomes from each sampled environment. Human-associated bacterial genomes are largely overrepresented in our dataset. As expected from aligning spacers to phage genomes of the human microbiome, CRISPR-Cas9 loci from the human microbiome have the highest number of matches per spacer. CRISPR-Cas9 loci identified in genomes from microbial isolates retrieved from the NCBI database were not included. Central line, median; box limits, upper and lower quartiles; whiskers, 1.5x interquartile range.

Supplementary Figure 2

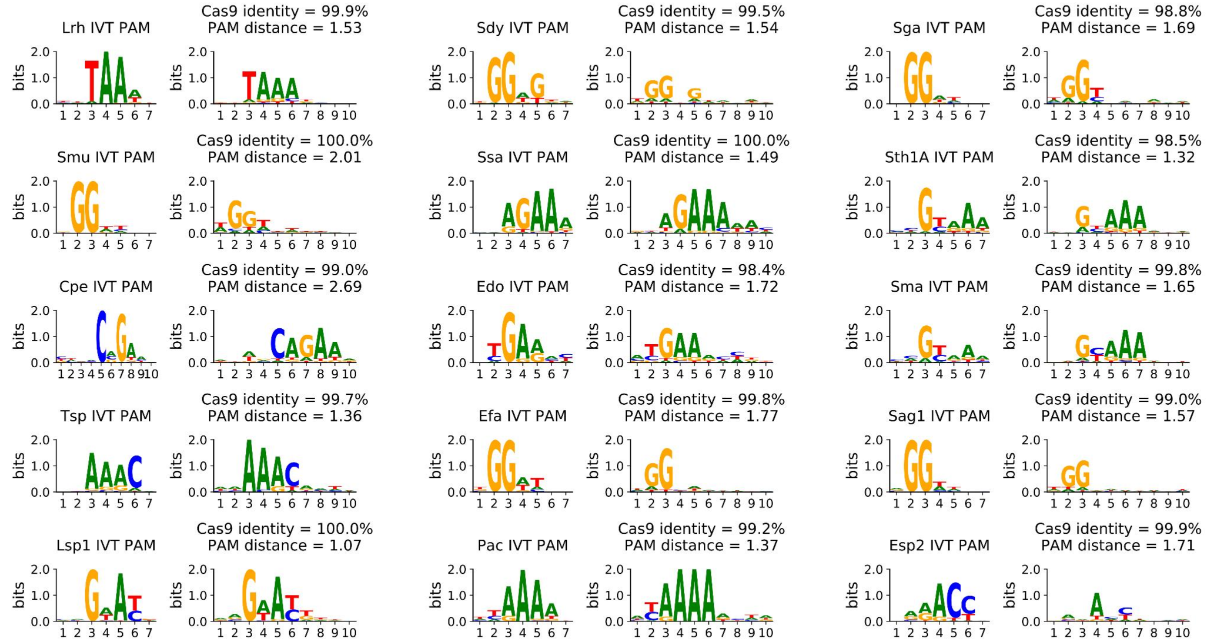

Supplementary Figure 2: Predicted and experimentally identified PAM logos analyzed for selected Cas9s reported by Gasiunas *et al.* [18]. For each indicated Cas9 the *in vitro* (IVT) experimentally derived (left) and *in silico* predicted (right) logos are reported. Percentages of sequence identity and measure of the distance between predicted and true PAMs (see **Methods** in main text) are reported for each Cas9.

Supplementary Figure 3

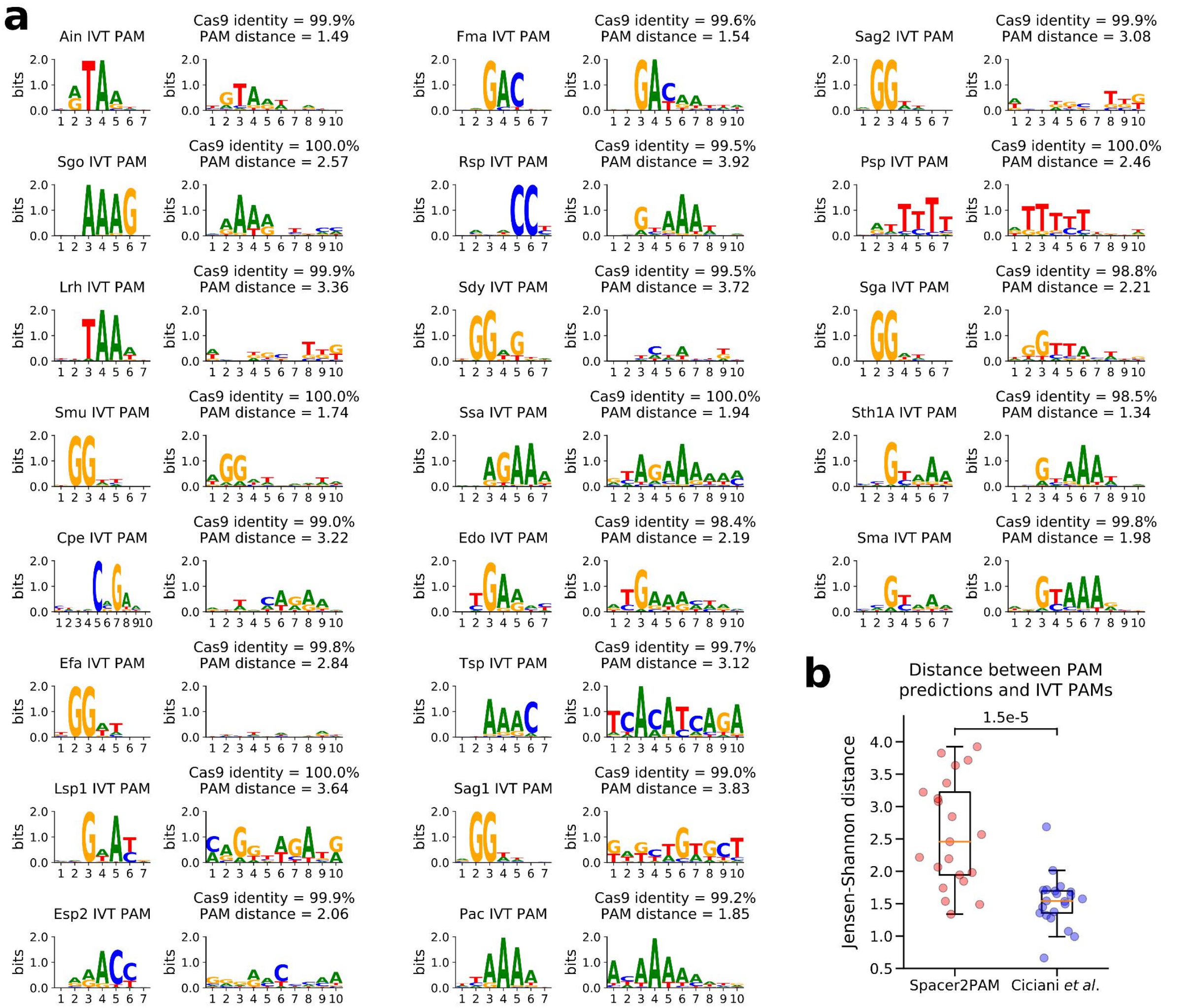

Supplementary Figure 3: Spacer2PAM predictions and experimentally identified PAM logos for selected Cas9s reported by Gasiunas *et al.* [18]. a) For each indicated Cas9 the *in vitro* (IVT) experimentally derived logo (left) and the logo predicted with Spacer2PAM (right) are reported. Percentages of sequence identity and measure of the distance between predicted and true PAMs (see **Methods** in main text) are reported for each Cas9. b) Boxplot of distance between *in vitro* and predicted PAMs generated by our method and Spacer2PAM for 21 selected Cas9s reported by Gasiunas *et al.* Our method generates more accurate predictions, with a statistically significant difference (p-value = 1.5e-5, two-sided Welch's t-test). Central

line, median; box limits, upper and lower quartiles; whiskers, 1.5x interquartile range; n=21 independent PAMs.

Supplementary Figure 4

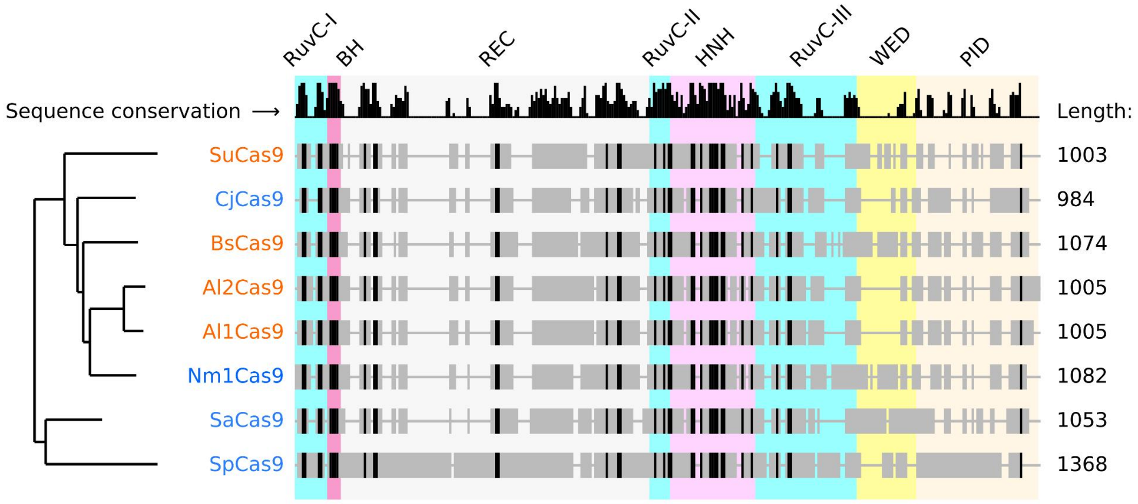

Supplementary Figure 4: Protein sequence alignments of newly discovered and described Cas9s. Phylogenetic tree of selected Cas9 proteins mostly used for genome editing applications (blue) and newly characterized Cas9 proteins (orange). Protein alignments: grey aligned protein sequences, black conserved sequences, colored conserved domains. Length: number of amino acids.

Supplementary Figure 5

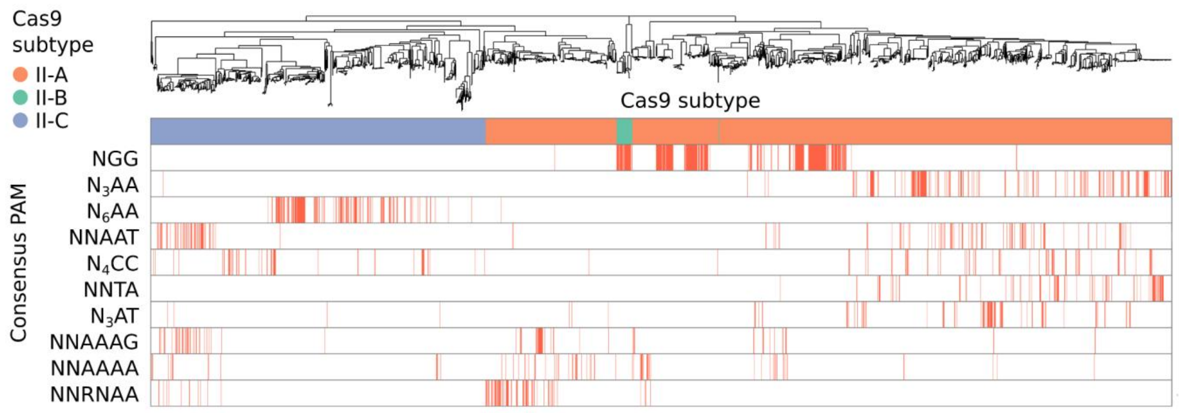

Supplementary Figure 5: Associations between the 10 most abundant PAM clusters and the Cas9 phylogenetic tree. The distribution of PAM clusters along the Cas9 phylogenetic tree is not random and each cluster is associated with specific clades of Cas9 proteins (statistically significant association, $p<0.001$ for each cluster, computed using a two-sided Fisher's exact test, with Monte Carlo simulation with 100,000 replicates and multiple hypothesis testing adjustment using the Benjamini-Hochberg correction, see **Methods**).

Supplementary Figure 6

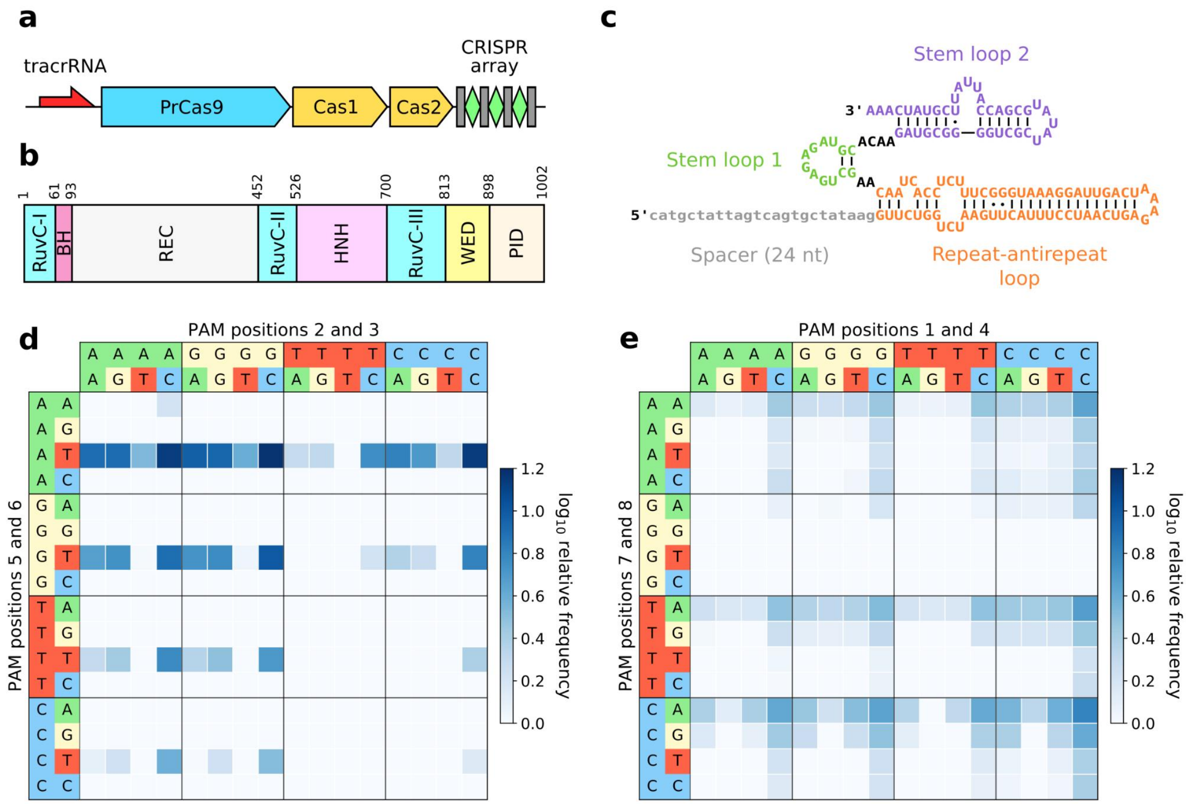

Supplementary Figure 6: PrCas9 identified for using as PAM the P23H-RHO mutation. a) Organization of the PrCas9 CRISPR-Cas locus. b) Predicted protein domain organization of PrCas9. c) Structure of the single guide RNA (sgRNA) of PrCas9. The repeat-antirepeat loop is followed by a short stem loop (Stem loop 1) and by a Rho-independent transcription terminator (Stem loop 2 and short poly-A tail). d) and e) PAM heatmap for PrCas9, showing the nucleotide preference for positions 2, 3, 5 and 6 (positions with conserved bases) and 1, 4, 7 and 8 (positions with non-conserved bases). Positions shown in e) do not exhibit strong preferences for any base and do not contribute to the PAM. The preferred PAM is NRVNRT, V = A, C or G; R = G or A.

Supplementary Figure 7

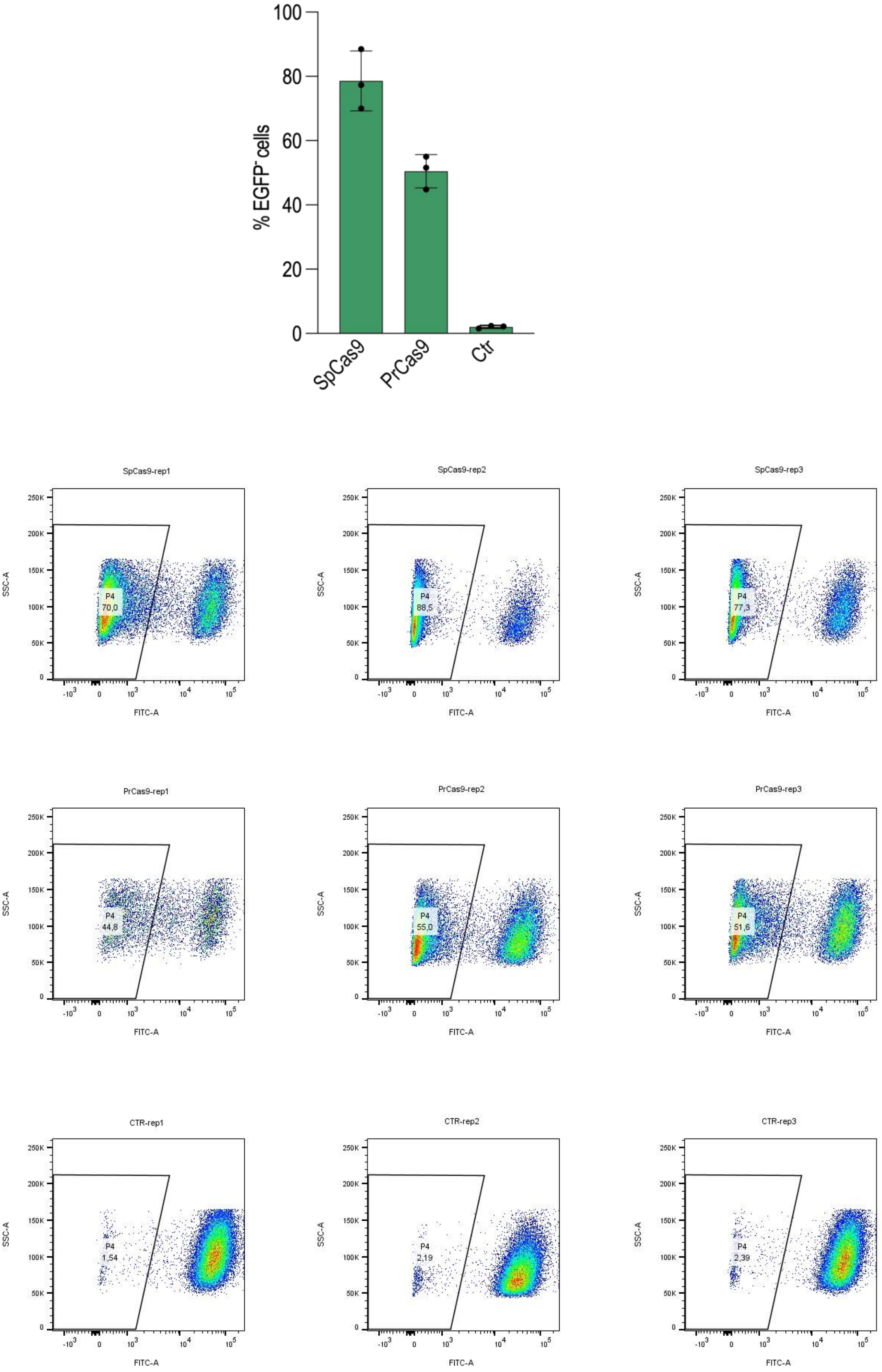

Supplementary Figure 7: Editing activity of PrCas9 in mammalian cells. The editing activity of PrCas9 was evaluated with an EGFP disruption assay by measuring through flow cytometry the loss of fluorescence of U2OS.EGFP cells transfected with SpCas9, PrCas9 or with a control plasmid (Ctr - no spacer plasmid) (upper image). Gating strategy relative to flow cytometry data shown above (bottom image); n=3 each, mean ± SD, biologically independent samples.

SUPPLEMENTARY METHODS

EGFP disruption assay

The assay to measure PrCas9 editing activity was performed using the U2OS.EGFP cells (a kind gift of Claudio Mussolino, University of Freiburg) carrying a single integrated copy of an EGFP reporter gene. U2OS.EGFP cells were cultured in DMEM (Life Technologies) supplemented with 10% FBS (Life Technologies), 2 mM GlutaMax (Life Technologies) and penicillin/streptomycin (Life Technologies) at 37°C and 5% CO_2 in a humidified atmosphere. Cells tested mycoplasma negative (PlasmoTest, Invivogen). To perform the experiments, 200,000 U2OS.EGFP cells were nucleofected with 1 ug of pX-PrCas9/pX-SpCas9 plasmid bearing a gRNA designed to target EGFP (**Supplementary Table 6**) or a non-targeting spacer (Ctr) using the 4D-Nucleofector™ X Kit (Lonza), DN100 program, according to the manufacturer's protocol. After electroporation, cells were plated in a 96-well plate. After 48 hours cells were expanded in a 24-well plate. EGFP knock-out was analyzed 4 days after nucleofection using a BD FACSCanto (BD) flow cytometer. Data was analyzed with FloJo v10.

**Supplementary Table 1:** Description of previously published PAM prediction methods					
**Method**	Spacer2PAM	Vink *et al.*	CASPERpam	SPAMALOT	CRISPRTarget
**PMID**	35258601	34593010	30076736	30397647	23492433
**Year**	2022	2021	2018	2018	2013
**Code publicly available**	Yes	No	Yes	No	No (Web Service)
**Code actively maintained**	Yes	N/A	No	N/A	Yes
**Spacer alignment databases**	NCBI nucleotide collection (nr/nt)	NCBI nucleotide database, environmental nucleotide database, PHASTER, Mgnify, IMG/M, IMG/Vr, HuVirDb, HMP database and data from Pasolli et al.	13,885 viral assemblies, 11 218 plasmid assemblies and 129,209 prokaryotic assemblies from NCBI databases	53 *Streptococcus* phages	ACLAME genes, Genbank-Environmental, Genbank-Phage, RefSeq-Archaea, RefSeq-Plasmid, RefSeq-Viral, IMGVR, Islandviewer, HuVirDB, PHAST, CAMERA selection

**Supplementary Table 2.** PrCas9 amino acid sequence.
MKMQDSVSKMKYRLGIDLGTTSLGWAMLRLDEQNEPYAVIRAGVRIFNNGRDPKTEASL<br>AVARRLARQQRRTRDRKIRRKERLIGELVDMGFFPKDPVKRRQLASLDPFKLRTEALDRA<br>LSPEEFARAIFHLARRRGFKSNRKTDSGDTESSKMKEAIKRTLNELQNKGFRTVGEWLN<br>MRHQQRLGTRSRIKNVPTGSGKQTTAYDFYLNRFMIEYEFDRIWEKQSQMNPGLFTNER<br>KAILKDIIFYQRPLRPVEPGRCTFMPDNPRAPLALPQQQDFRIYQEVNNLRKIDPTSLLEVN<br>LTLPERDRIVELLQRKPALTFDAVRKALCFNGTFNLEGENRSELKGNLTNCALAKKKLFGE<br>SWYSFDAHKRFEIVEHLLQEESEENLVSWLQKECNLSEEYAKNVASVRLPAGYGALCQE<br>ALDLILPYLKAEVITYDKAVQKAGMNHSELTLAQETGEILPELPYYGQYLKRHVGFGTGKP<br>EDSAEKRYGKIPNPTVHIALNQLRTVVNALIRRYGKPTQIVIELARELKQNKKAKDQYRIEM<br>NHNQNRNERIRADISMILGINPENVKRKDIEKQILWEELNLKDATARCCPYSGKQISAEMLF<br>TDEVEIDHILPFSRTLDDSKNNKVVCIREANRIKGNRTPWEARKDFEKRGWSVEAMTARA<br>QAMPKAKRFRFAEDGYKVWLKDFDGFEARALTDTQYMSRVAREYLQLICPGQTWSVPG<br>QLTGMLRRFLGLNDILGVNGEKNRDDHRHHAVDACVIALTDRSMLQRISTASARAENKHL<br>TRLLESFPAPWATFYEHVTRAVKSICVSHKPEHAYQGAMNEQTAYGLRPDGYVKYRQNG<br>KVEHKKLNVIPQVSVKGTWRHGLNSDGSLKAYKGLKGGSNFCIEIVMGEGGRWEGDVIT<br>TYEAYQIVRAKGEAALYGSVSRSGKPLVMRLMQKDIVEMTLADGRCKMLLYIITQNKQMF<br>FYRIENAGGGREDVSKRPGSLQKALAKKIIVSPIGDFRKEKL*

Supplementary Table 3. Description of metagenomic datasets from which 54,169 additional MAGs were retrieved

Study ID	PMID / DOI	Environment	Number of samples	Number of MAGs
PaoliL_2021	10.1101/2021.03.24.436479	Ocean	6261	32366
BeresfordJonesBS_2021	34971560	Mice gut	1043	18733
HeroldM_2020	33077707	Wastewater	787	2098
AlbaneseD_2021	33741058	Antarctic desert	33	562
LeechJ_2020	33172966	Fermented food	70	228
LandisEA_2021	33496265	Bakery	43	107
WylensekD_2020	33319778	Pig gut	38	75
KastmanEK_2016	27795388	Dairy	19	56
PfeferT_xxxx	-	Dairy	35	54
LiZ_2018	30166168	Fermented foods	11	49
ArikanM_2020	31957879	Fermented foods	12	35
PatroJN_2016	27303722	Probiotics	10	33
ZhaoCC_2020	32247457	Fermented foods	5	32
ChaconVargasK_2020	32934253	Fermented foods	8	32
PothakosV_2020	10.1016/j.crbiot.2020.02.001	Fermented foods	10	29
PasolliE_2020	32451391	Dairy;Probiotics	17	28
KumarJ_2019	31428064	Fermented foods	4	21
VerceM_2019	30918501	Fermented foods	6	21
YulandiA_2020	33308279	Fermented foods	2	20
EscobarZepedaA_2016	27052710	Dairy	1	18
SulaimanJ_2014	25400624	Brine	7	18

DuruIC_2018	29803134	Dairy	6	17
Ferrocinol_2018	29196291	Fermented foods	11	17
DuR_2020	32276974	Alcohol	3	16
LiZ_2019	31500701	Fermented foods	6	13
PorcellatoD_2016	10.1016/j.idairyj.2016.05.005	Dairy	12	13
EinsonEJ_2018	30171008	Fermented foods;Fruit and Vegetables	4	12
LordanR_2019	10.1016/j.jff.2019.01.029	Dairy	5	11
SalvettiE_2016	27445999	Fruit and Vegetables	2	11
LeonardSR_2016	27930729	Fruit and Vegetables	7	9
DeRoosJ_2020	32765478	Alcohol	4	8
YasirM_2020	33233218	Dairy	2	3
SomervilleV_2019	31238873	Dairy	1	2
CrovadoreJ_2017	28572315	Other	2	2

**Supplementary Table 4**. Sequences of the primers used for NGS library preparation in the *in vitro* PAM assay	
Primer name	Sequence (5’ → 3’)
F4a	TCGTCGGCAGCGTCAGATGTGTATAAGAGACAGCTGCTGAACCGCTCTTCCGATC
F4b	TCGTCGGCAGCGTCAGATGTGTATAAGAGACAGTAAGACTGCTGAACCGCTCTTCCGATC
F4c	TCGTCGGCAGCGTCAGATGTGTATAAGAGACAGGCTAGACCTAATGTGATCTGCTGAACCGCTCTTCCGATC
R3	GTCTCGTGGGCTCGGAGATGTGTATAAGAGACAGTCTGCGTTCTGATTTAATCTGTATCAGGC

Supplementary Table 5. Distance between PAM predictions and IVT PAMs for 16 Cas9 proteins characterized by Gasiunas *et al.* that have a prediction at every clustering identity level.

	**% identity clustering**					
**Cas9 ID**	**100**	**99**	**98**	**97**	**96**	**95**
**Smu**	2.013	2.013	2.013	2.013	2.013	2.013
**Psp**	0.666	0.661	0.661	0.661	0.662	0.661
**Lrh**	1.535	1.535	1.535	1.535	1.563	1.563
**Efa**	1.928	1.769	1.766	1.766	1.766	1.766
**Ain**	1.512	0.991	0.991	0.972	0.972	0.972
**Rsp**	1.549	1.263	1.276	1.276	1.276	1.276
**Sag1**	1.569	1.565	1.574	1.574	1.577	1.532
**Lsp1**	1.065	1.065	1.073	1.073	1.073	1.073
**Pac**	2.571	2.571	1.374	1.261	1.205	1.302
**Tsp**	1.411	1.355	1.355	1.355	1.355	1.355
**Ssa**	1.495	1.495	1.495	2.124	2.115	1.72
**Sma**	2.044	1.653	1.653	1.603	1.499	1.777
**Sag2**	1.62	1.627	1.627	1.625	1.625	1.625
**Cpe**	2.768	3.693	2.687	2.71	2.685	2.691
**Sdy**	1.902	1.542	1.542	1.542	1.535	1.539
**Sga**	2.299	2.299	1.695	1.695	1.695	1.695
**Median**	1.595	1.554	1.539	1.558	1.549	1.551

Supplementary Table 6. Sequences of the oligonucleotides used for spacers cloning in the expression plasmids.

**Plasmid**	**Protospacer**	**Target (*)**	**Oligonucleotide 1 (5'-3')**	**Oligonucleotide 2 (5'-3')**
pX-SpCas9-sgRNA-GFPB	CCGGCAAGCTGCCCGTGCCC (**)	ccaCCGGCAAGCTGCCCGTGCCC**TGG**ccc	CACCGCCGGCAAGCTGCCCGTGCCC	AAACGGGCACGGGCAGCTTGCCGGC
pX-PrCas9-sgRNA-GFP	GCCCGAAGGCTACGTCCAGGAGCG	catGCCCGAAGGCTACGTCCAGGAGCG**CACCAT**ctt	CACCGCCCGAAGGCTACGTCCAGGAGCG	GAACCGCTCCTGGACGTAGCCTTCGGGC
pX-PrCas9-sgRNA-RHO-P23H	CAGCCAGGTAGTACTGTGGGTACT	gctCAGCCAGGTAGTACTGTGGGTACT**CGAAGG**ggc (RHO WT)	CACCGCAGCCAGGTAGTACTGTGGGTACT	GAACAGTACCCACAGTACTACCTGGCTGC
		gctCAGCCAGGTAGTACTGTGGGTACT**CGAAGT**ggc (RHO P23H)		

(*) PAM is in bold. Nucleotides around the target site are in lowercase.

(**) Validated gRNA in Casini, A., Olivieri, M., Petris, G. et al. A highly specific SpCas9 variant is identified by in vivo screening in yeast. Nat Biotechnol 36, 265–271 (2018). https://doi.org/10.1038/nbt.4066
