# Supplementary material for: Automated identification of sequence-tailored Cas9 proteins using massive metagenomic data
Source: Nat Commun. 2022 Oct 29;13:6474. doi: 10.1038/s41467-022-34213-9 (PMC9617884; doi:10.1038/s41467-022-34213-9)
Supplement: Supplementary file 2 — Reporting Summary [file 41467_2022_34213_MOESM2_ESM.pdf]

nature portfolio

Corresponding author(s): Anna Cereseto, Nicola Segata

Last updated by author(s): Oct 6, 2022

Reporting Summary

Nature Portfolio wishes to improve the reproducibility of the work that we publish. This form provides structure for consistency and transparency in reporting. For further information on Nature Portfolio policies, see our Editorial Policies and the Editorial Policy Checklist.

Statistics

For all statistical analyses, confirm that the following items are present in the figure legend, table legend, main text, or Methods section.

n/a	Confirmed	
☐	☒	The exact sample size (*n*) for each experimental group/condition, given as a discrete number and unit of measurement
☐	☒	A statement on whether measurements were taken from distinct samples or whether the same sample was measured repeatedly
☐	☒	The statistical test(s) used AND whether they are one- or two-sided<br>*Only common tests should be described solely by name; describe more complex techniques in the Methods section.*
☒	☐	A description of all covariates tested
☐	☒	A description of any assumptions or corrections, such as tests of normality and adjustment for multiple comparisons
☐	☒	A full description of the statistical parameters including central tendency (e.g. means) or other basic estimates (e.g. regression coefficient) AND variation (e.g. standard deviation) or associated estimates of uncertainty (e.g. confidence intervals)
☐	☒	For null hypothesis testing, the test statistic (e.g. *F*, *t*, *r*) with confidence intervals, effect sizes, degrees of freedom and *P* value noted<br>*Give P values as exact values whenever suitable.*
☒	☐	For Bayesian analysis, information on the choice of priors and Markov chain Monte Carlo settings
☒	☐	For hierarchical and complex designs, identification of the appropriate level for tests and full reporting of outcomes
☒	☐	Estimates of effect sizes (e.g. Cohen's *d*, Pearson's *r*), indicating how they were calculated

Our web collection on statistics for biologists contains articles on many of the points above.

Software and code

Policy information about availability of computer code

Data collection	Next-generation sequencing data was collected with Illumina MiSeq.
Data analysis	Analyses were preformed using custom code in Python (3.7) and R (4.1.0). Additional software used: metaSPAdes (3.10.1), MEGAHIT (1.1.1), MetaBAT2, ViromeQC (1.0), TrimGalore (0.4.4), Bowtie2 (2.4.1), mash (2.0), blastn (2.5.0 and 2.6.0), VSEARCH, CRISPRCasTyper (1.5.0), usearch (11.0.667), MUSCLE (3.8.31), Logomaker (0.8), RNIE, mafft (7.490), FastTree (2.1.11), TreeCluster (1.0.3), Spacer2PAM (0.0.0.9000), FloJo (v10). Custom code used in this study is available at: https://github.com/Matteo-Ciciani/PAMpredict (PAM prediction tool) and https://github.com/Matteo-Ciciani/PAM_prediction_analysis (additional code used to analyze PAM predictions).

For manuscripts utilizing custom algorithms or software that are central to the research but not yet described in published literature, software must be made available to editors and reviewers. We strongly encourage code deposition in a community repository (e.g. GitHub). See the Nature Portfolio guidelines for submitting code & software for further information.

Data

Policy information about availability of data

All manuscripts must include a data availability statement. This statement should provide the following information, where applicable:

- Accession codes, unique identifiers, or web links for publicly available datasets
- A description of any restrictions on data availability
- For clinical datasets or third party data, please ensure that the statement adheres to our policy

The data that support the findings of this study are available from the the material associated with https://doi.org/10.1101/2022.08.22.504593 .

Human research participants

Policy information about studies involving human research participants and Sex and Gender in Research.

Reporting on sex and gender	N/A
Population characteristics	N/A
Recruitment	N/A
Ethics oversight	N/A

Note that full information on the approval of the study protocol must also be provided in the manuscript.

Field-specific reporting

Please select the one below that is the best fit for your research. If you are not sure, read the appropriate sections before making your selection.

☒ Life sciences ☐ Behavioural & social sciences ☐ Ecological, evolutionary & environmental sciences

For a reference copy of the document with all sections, see nature.com/documents/nr-reporting-summary-flat.pdf

Life sciences study design

All studies must disclose on these points even when the disclosure is negative.

Sample size	No stastistical methods were used to predetermine sample size. No sample size assessment was required since only the editing activity of PrCas9 was assessed on GFP and RHO genes.
Data exclusions	No data were excluded.
Replication	All experiments were independently performed three times.
Randomization	Randomization was not relevant for this study since we are not analysing or comparing populations/treatments.
Blinding	Blinding was not relevant to this study since there are no treatment comparison experiments.

Reporting for specific materials, systems and methods

We require information from authors about some types of materials, experimental systems and methods used in many studies. Here, indicate whether each material, system or method listed is relevant to your study. If you are not sure if a list item applies to your research, read the appropriate section before selecting a response.

Materials & experimental systems

n/a	Involved in the study	
☒	☐	Antibodies
☐	☒	Eukaryotic cell lines
☒	☐	Palaeontology and archaeology
☒	☐	Animals and other organisms
☒	☐	Clinical data
☒	☐	Dual use research of concern

Methods

n/a	Involved in the study	
☒	☐	ChIP-seq
☐	☒	Flow cytometry
☒	☐	MRI-based neuroimaging

Eukaryotic cell lines

Policy information about cell lines and Sex and Gender in Research

Cell line source(s)	HEK293T/17 cells were obtained from ATCC and U2OS_EGFP were kindly gifted by Dr. Mussolino (no commercial source). All details about HEK293T/17 and U2OS_EGFP are listed in Methods and Supplementary Methods.
Authentication	HEK293T/17 and U2OS_EGFP were not authenticated but validated for carrying the editing target sequence
Mycoplasma contamination	HEK293T/17 cells are periodically tested for mycoplasma. We used mycoplasma negative cells
Commonly misidentified lines (See ICLAC register)	No commonly misidentified cell line has been used

Flow Cytometry

Plots

Confirm that:

☒ The axis labels state the marker and fluorochrome used (e.g. CD4-FITC).

☒ The axis scales are clearly visible. Include numbers along axes only for bottom left plot of group (a 'group' is an analysis of identical markers).

☒ All plots are contour plots with outliers or pseudocolor plots.

☒ A numerical value for number of cells or percentage (with statistics) is provided.

Methodology

Sample preparation	Samples were run without fixation after cells wash in PBS
Instrument	FACS Canto Becton Dickinson
Software	the software provided by Becton Dickinson with the instrument and FloJo v10
Cell population abundance	No sorting was performed
Gating strategy	The gating strategy was defined using negative controls (cells non edited)

☒ Tick this box to confirm that a figure exemplifying the gating strategy is provided in the Supplementary Information.
